# Clinical Outcomes of Transjugular Intrahepatic Portosystemic Shunt Among Cirrhosis Patients With Recurrent Esophageal Variceal Bleeding

**DOI:** 10.7759/cureus.73101

**Published:** 2024-11-05

**Authors:** Muhammad Shafiq, Mehran A Khan, Shahryar Khan

**Affiliations:** 1 Internal Medicine, University of Kansas Medical Center, Kansas City, USA; 2 Medicine, Nowshera Medical College, Nowshera, PAK

**Keywords:** esophageal variceal banding, mortality, outcomes, readmission, recurrent esophageal variceal bleeding, tips

## Abstract

Background

Transjugular intrahepatic portosystemic shunt (TIPS) has been shown to reduce the risk of rebleeding among patients with recurrent esophageal variceal bleeding. However, the impact of TIPS on survival remains uncertain. This study took on this challenge to determine if TIPS has any impact on all-cause inpatient mortality during the hospitalization in which it is performed and if it impacts all-cause 30-day readmission rates when compared to patients who only undergo esophageal variceal banding (EVB) for recurrent esophageal variceal bleeding.

Methods

This was a retrospective cohort study using the Healthcare Cost and Utilization Project - National Readmission Database 2019. All adult patients who had a diagnosis of cirrhosis, were hospitalized once for esophageal variceal bleeding requiring EVB in 2019, and then were hospitalized again later in the year for recurrent esophageal variceal bleeding requiring either TIPS or EVB were included. This second hospitalization in which patients either received TIPS or EVB only was considered as the index hospitalization. Patients with missing data and patients who were discharged in December were excluded. The primary outcome was all-cause 30-day readmission rates among patients who either received TIPS or EVB only. The secondary outcomes included: (i) incidence of all-cause inpatient mortality; and (ii) length of hospital stay during index hospitalization.

Patients who underwent TIPS were matched with patients who underwent EVB only on age, sex, and baseline comorbidities. After propensity score matching, survival analysis was performed to compare the all-cause 30-day readmission rates after the index hospitalization, between patients who either received TIPS or EVB only during the index hospitalization. χ2 test was used to compare the inpatient mortality. As the data did not have a normal distribution, the Wilcoxon signed-rank test was used to compare the length of index hospitalization between patients who either received TIPS or EVB only. The alpha criterion was set at 0.05 for all statistical tests.

Results

This study found no difference in all-cause 30-day readmission rates between patients who underwent either TIPS or EVB only for recurrent esophageal variceal bleeding (hazard ratio: 1.24, 95%CI: 0.73-2.12, P = 0.4). In the exploratory analysis, it was noted that the rate of recurrent esophageal variceal bleeding, among patients who were readmitted within 30 days was lower in the TIPS group (13.3%) when compared to the EVB group (50%) with a risk ratio of 0.27 (95%CI: 0.10-0.72, P = 0.003). Although it was not statistically significant, the inpatient mortality rate during the index hospitalization appeared to be lower in the TIPS group when compared to the EVB group (4.2% *vs* 10.08%, respectively). Patients who underwent TIPS were hospitalized three days longer than patients who underwent EVB only.

Conclusion

TIPS does not reduce all-cause 30-day readmission rates but is associated with reduced 30-day readmission rates secondary to recurrent esophageal variceal bleeding. TIPS shows a modest survival advantage during index hospitalization when compared to EVB only.

## Introduction

Cirrhosis was the most common cause of liver-related deaths in 2017, with an estimated 1.32 million deaths globally [1,2]. Important complications of cirrhosis include the development of ascites, hepatic encephalopathy, and esophageal varices [3]. In particular, the cumulative incidence of esophageal varices increases over time with reported incidence of 44% and 53% at 10 and 20 years, respectively [4]. When esophageal varices bleed, they represent a life-threatening medical emergency with a reported six-week mortality rate of 16-22% despite endoscopic interventions [5,6]. Therefore, timely management of acute variceal bleeding is of paramount importance [7].

In the event of initial acute variceal bleeding (besides fluid resuscitation, use of vasoactive medications to ensure appropriate hemodynamics for adequate organ perfusion, antimicrobial prophylaxis, and abdominal imaging), the practice guidelines of the American Association for the Study of Liver Disease also recommends esophagogastroduodenoscopy (EGD) within 12 hours of presentation to determine the source of bleeding and to ensure timely endoscopic intervention to stop bleeding such as esophageal variceal banding (EVB) or sclerotherapy [7]. Due to the risk of rebleeding, repeat EGD every two to four weeks with repeat EVB until complete obliteration of the varices is now the standard of care [7-9]. For decompensated cirrhosis patients who rebleed early despite secondary prophylaxis and EVB, a transjugular intrahepatic portosystemic shunt (TIPS) is then recommended [10]. Another major indication for TIPS includes refractory ascites [11].

Simonetti et al. in their Cochrane review have reported that the risk of rebleeding after TIPS is 17.3% compared to 43.2% in the endoscopic intervention group [12]. This suggests that TIPS is associated with a reduced risk of recurrent esophageal variceal bleeding. However, the impact of TIPS on survival remains uncertain [12]. To add and explore further, we also took on this challenge to determine if TIPS has any impact on all-cause inpatient mortality during the hospitalization in which it is performed and if it impacts all-cause 30-day readmission rates when compared to patients who only undergo EVB.

## Materials and methods

Study design

This was a retrospective cohort study using the Healthcare Cost and Utilization Project - National Readmission Database (HCUP-NRD) 2019. The International Classification of Diseases, 10th Revision (ICD-10) diagnostic codes were used to define the computable phenotypes for cirrhosis, esophageal variceal bleeding, EVB, TIPS procedure, baseline comorbidities, anticoagulation or comorbidities that may require anticoagulation, causes of cirrhosis, and complications of cirrhosis. Once the computable phenotypes were defined for the aforementioned elements, the "dplyr" package in R software version 4.3.3 (R Foundation for Statistical Computing, Vienna, Austria) was used to extract data from the HCUP-NRD 2019. Details regarding the exact definitions of the computable phenotypes and methods of their extraction from the HCUP-NRD 2019 are provided in the Appendices.

Data regarding readmissions, age, sex, month of admission, length of hospital stay, inpatient mortality, teaching status of the hospital, and hospital size were directly provided by the HCUP-NRD 2019.

Selection criteria

The inclusion criteria included all adult patients (aged 18 years and older) who had a diagnosis of cirrhosis, were hospitalized once for esophageal variceal bleeding requiring EVB in 2019, and then were hospitalized again later in the year for recurrent esophageal variceal bleeding requiring either TIPS or EVB only. Exclusion criteria included patients with missing data and patients who were discharged in December as for those patients, 30-day readmission outcomes could not be calculated because NRD does not link data from one year to another. 

Index hospitalization

Index hospitalization has been defined with the help of an example: Mr. A was diagnosed with cirrhosis and was hospitalized in February 2019 for esophageal variceal bleeding. During this first hospitalization, the patient received EVB. The patient was then readmitted again in May of 2019 for recurrent variceal bleeding. This second hospitalization for the recurrent esophageal variceal bleeding (despite EVB in the prior hospitalization) was defined as the index hospitalization. For Mr. A to be included in this study, he must have received either TIPS or repeat EVB during this index hospitalization. 

Groups

Once the final cohort was identified, patients were divided into two groups: those who received TIPS and those who received EVB only during the index hospitalization.

Outcomes

The primary outcome was all-cause 30-day readmission rates after the index hospitalization, among patients who either received TIPS or EVB only. The secondary outcomes included: (i) incidence of all-cause inpatient mortality during index hospitalization and (ii) length of hospital stay during index hospitalization among patients who either received TIPS or EVB only.

Statistical analysis

After the identification of the final cohort, patients who underwent TIPS during the index hospitalization were then matched with patients who underwent EVB only on age, sex, and baseline comorbidities, including congestive heart failure, ischemic heart disease, hypertension, hyperlipidemia, diabetes mellitus, chronic kidney disease/end-stage renal disease, chronic obstructive pulmonary disease/emphysema, and asthma. Since anticoagulation increases the risk of bleeding, these two groups were also matched on anticoagulation use. In addition, these two groups were also matched on conditions that may be a contraindication for TIPS (including pulmonary hypertension, tricuspid regurgitation, and polycystic liver disease). For instance, if a patient does not undergo TIPS because of severe pulmonary hypertension and then dies, it is likely because (i) the patient did not get TIPS, and (ii) the severe pulmonary hypertension likely also played an independent role. Propensity score matching (PSM) with the nearest neighbor method at a 1:1 ratio and without replacement was used for matching. The caliper was set at 0.1 for PSM.

After PSM was complete, survival analysis was performed to compare the all-cause 30-day readmission rates after the index hospitalization between patients who either received TIPS or EVB only (primary outcome). For survival analysis, a Kaplan‒Meier survival curve was constructed, and a Cox proportional hazards model was used to calculate the hazard ratio. The proportional hazards assumption was assessed using the Schoenfeld proportionality test.

The χ2 test was used to compare the inpatient mortality between the two groups during the index hospitalization. Both the mortality rate and risk ratio (RR) were calculated. As the duration of hospital stay did not have a normal distribution, the Wilcoxon signed-rank test was used to compare the length of index hospitalization between patients who either received TIPS or EVB only. 

Binomial regression and adjusted Cox proportional hazard models were used in exploratory analysis. Assumptions for these models were assessed and detailed in the result section. 

The alpha criterion was set at 0.05 for all statistical tests. Weighted analysis was not performed because this study focused on comparative analysis between two groups rather than calculating the national estimates. R software version 4.3.3 (R Foundation for Statistical Computing, Vienna, Austria) was used for data extraction, data cleaning, and analysis.

## Results

**Figure 1:**
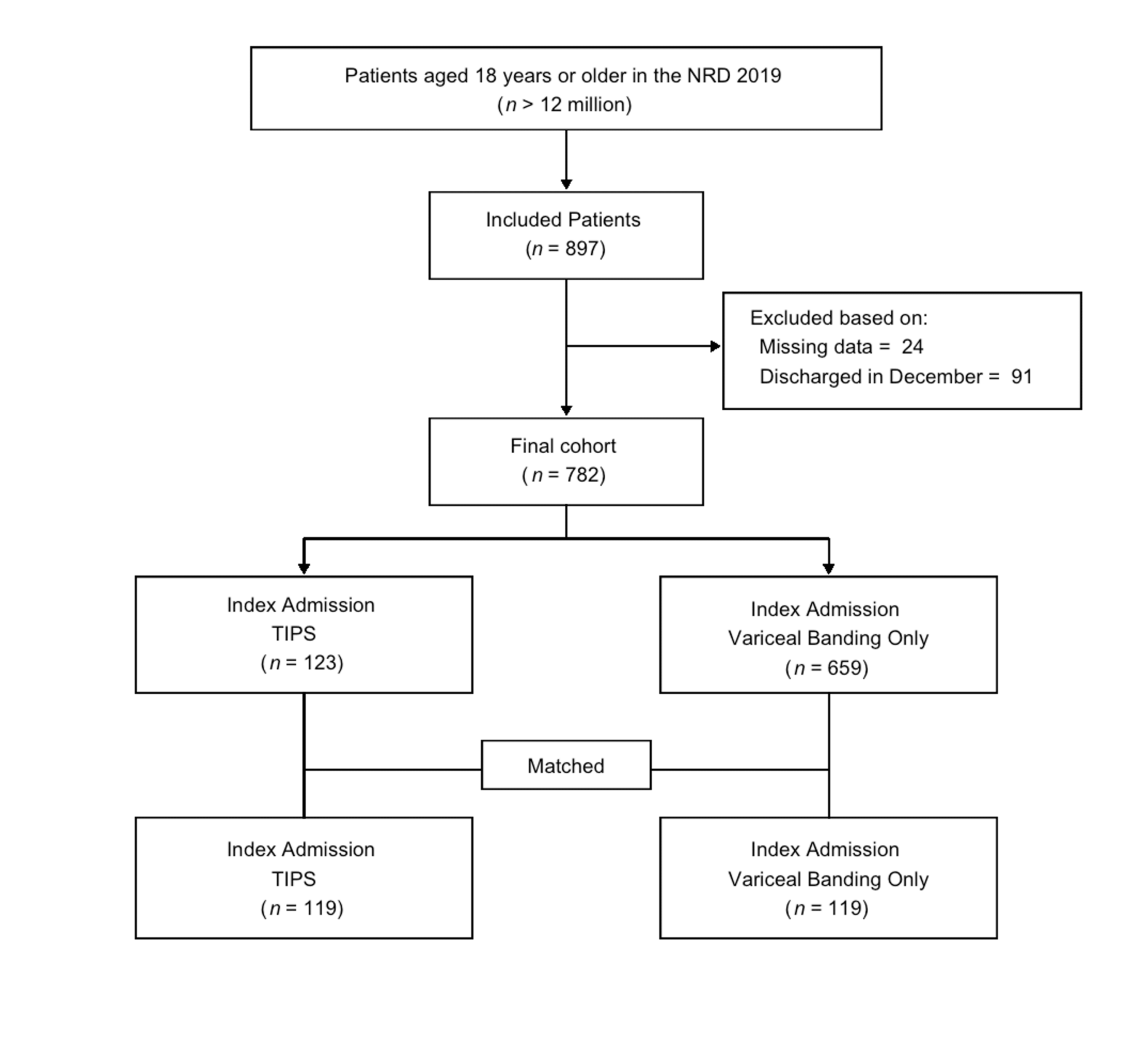
Flowchart of the cohort selection NRD: National Readmission Database; TIPS: transjugular intrahepatic portosystemic shunt

There are more than 12 million unique adult patients in the HCUP-NRD 2019. Of those patients, 782 patients were included in the final cohort, as illustrated in Figure 1. Once the final cohort was selected, patients who had undergone TIPS during the index hospitalization were then matched with patients who had undergone EVB only during the index hospitalization using PSM, as summarized in Table 1.

**Table 1:** Demographics and baseline characteristics of patients who underwent TIPS vs variceal banding only during the index hospitalization, before and after propensity score matching TIPS: transjugular intrahepatic portosystemic shunt; CHF: congestive heart failure; CKD: chronic kidney disease; COPD: chronic obstructive pulmonary disease; DM: diabetes mellitus; ESRD: end-stage renal disease; HLD: hyperlipidemia; HTN: hypertension; IHD: ischemic heart disease; SD: standard deviation

Demographics and baseline comorbidities	Before matching		After matching	
	TIPS (n = 123)	Variceal Banding (n = 659)	TIPS (n = 119)	Variceal Banding (n = 119)
Age in years, mean±SD	54.47±12.84	54.55±11.95	54.45±12.75	54.62±12.58
Sex = Male, n (%)	92 (74.8)	475 (72.1)	89 (74.8)	84 (70.6)
CHF = Yes, n (%)	11 (8.9)	62 (9.4)	11 (9.2)	11 (9.2)
IHD = Yes, n (%)	19 (15.4)	71 (10.8)	17 (14.3)	18 (15.1)
HTN = Yes, n (%)	74 (60.2)	390 (59.2)	71 (59.7)	75 (63.0)
HLD= Yes, n (%)	39 (31.7)	136 (20.6)	36 (30.3)	36 (30.3)
DM = Yes, n (%)	43 (35.0)	208 (31.6)	41 (34.5)	42 (35.3)
CKD/ESRD = Yes, n (%)	19 (15.4)	107 (16.2)	19 (16.0)	23 (19.3)
COPD/Emphysema = Yes, n (%)	17 (13.8)	93 (14.1)	16 (13.4)	18 (15.1)
Anticoagulation = Yes, n (%)	26 (21.1)	102 (15.5)	24 (20.2)	25 (21.0)

Patients were also matched on asthma, pulmonary hypertension, tricupid regurgitation, and polycystic liver disease. However, this data have not been provided in Table 1 because the count for these comorbidities was less than 11 and HCUP does not recommend to provide such data in tableted form. 

Primary outcome

**Figure 2:**
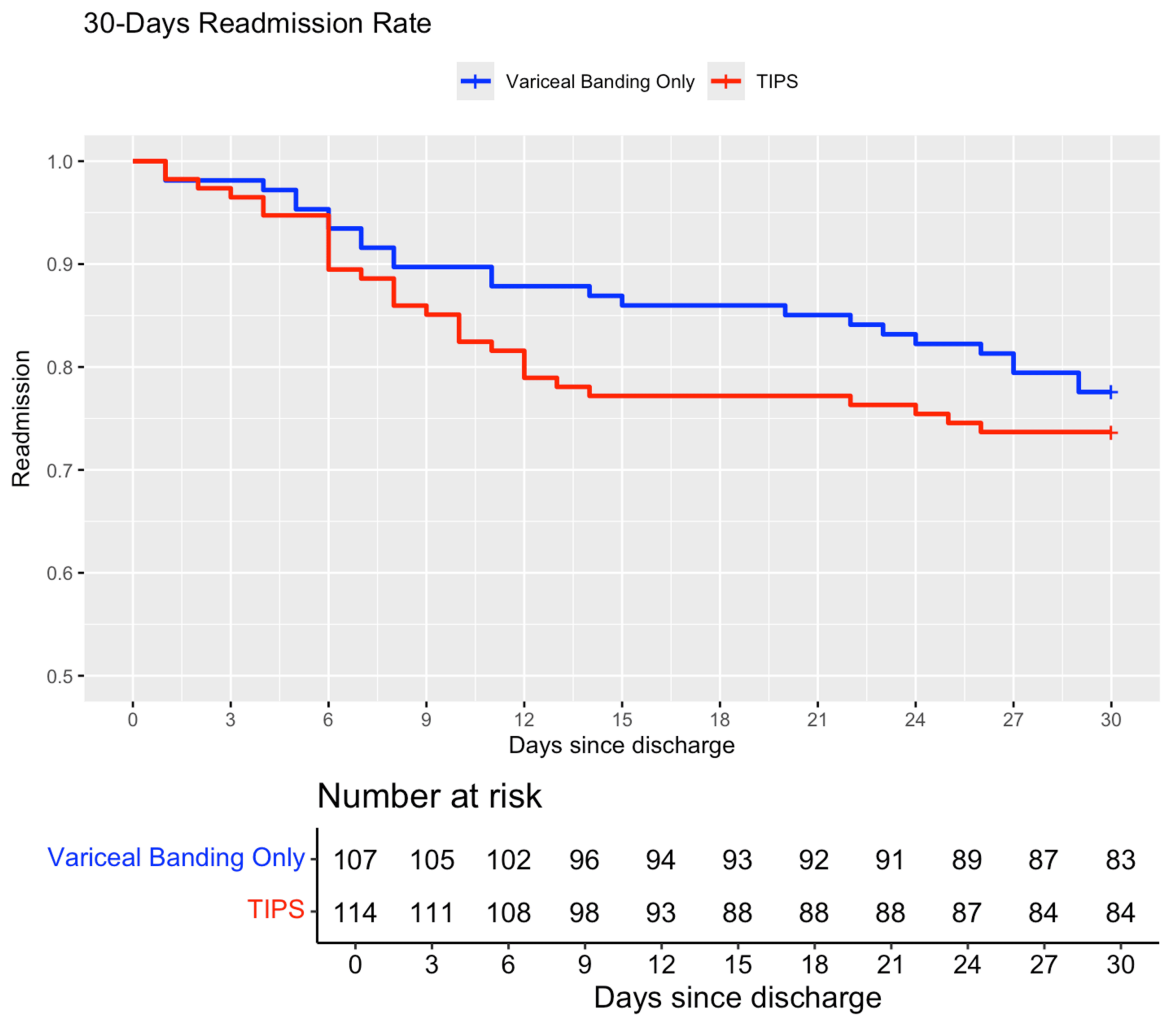
Kaplan-Meier survival curve for 30-day readmission outcome between patients who underwent TIPS vs variceal banding only during the index hospitalization TIPS: transjugular intrahepatic portosystemic shunt

After PSM was complete, survival analysis was performed. Of 119 patients in the TIPS matched-group, 114 patients were discharged alive and were included in the survival analysis. Of 119 patients in the EVB matched-group, 107 patients were discharged alive and were included in the survival analysis. As shown in Figure 2, the Kaplan-Meier survival plot does not show any significant deviation in 30-day readmission rates between patients who underwent TIPS vs EVB only during the index hospitalization.

Out of 114 patients who received TIPS and were discharged alive, 30 (26.32%) were readmitted within 30 days. Out of 107 patients who underwent EVB only during index hospitalization and were discharged alive, 24 (22.43%) patients were readmitted within 30 days. The hazard ratio for the all-cause 30-day readmission rate between patients who underwent TIPS vs EVB only during the index hospitalization was 1.24 (95%CI: 0.73-2.12, P = 0.4). Hence, there was no significant difference between the two groups in regard to all-cause 30-day readmission rates. The proportional hazards assumption was met (Schoenfeld proportionality test, P > 0.05).

Secondary outcomes

After PSM was complete, all-cause inpatient mortality during index hospitalization was noted to be 4.2% among patients who underwent TIPS vs 10.08% in EVB (RR: 0.42; confidence interval: 0.15 - 1.15; p-value: 0.078). Patients who underwent TIPS were hospitalized for a longer duration (with a median hospital length of stay of seven days with an interquartile range (IQR) of five days) than patients who underwent EVB only (with a median hospital length of stay of four days with IQR of three days), with a median difference of three days (95%CI: 1.99-3.00, P < 0.001).

Exploratory analysis

Among all patients that were matched (n = 238), alcohol use was the most common cause of cirrhosis (n = 141, 59.24%), followed by chronic hepatitis C infection with simultaneous use of alcohol (n = 42, 17.65%) and non-alcoholic steatohepatitis (n = 19, 7.98%). Among matched patients (n = 238), ascites was the most common complication in both TIPS (n= 77, 64.7%) and EVB group (n= 56, 47.1%). Blood transfusion was noted in 43.7% (n=52) of TIPS patients vs 42% (n= 50) of EVB patients.

Among matched patients (n = 238) in this study, binomial regression model showed a significant association between inpatient mortality and encephalopathy (RR: 4.83, 95%CI: 1.5-11.5, P = 0.002) as well as hepatorenal syndrome (RR: 6.15, 95%CI: 2.06-14.09, P < 0.001) during index hospitalization. The binomial regression model did not show any association between inpatient mortality and age, sex, ascites, spontaneous bacterial peritonitis, hepatocellular carcinoma, red blood cell transfusion, teaching status of a hospital, or hospital size during index hospitalization. The binomial regression model met all assumptions, including the assumption of no multicollinearity, and the model appeared to fit the data adequately (Hosmer‒Lemeshow goodness-of-fit test, P > 0.05).

Among matched patients who were discharged alive (n = 221) in this study, the adjusted Cox-proportional hazard model showed a statistically significant association between age and 30-day readmission rate after the index hospitalization. Ascites, spontaneous bacterial peritonitis, encephalopathy, hepatorenal syndrome, hepatocellular carcinoma, red blood cell transfusion, teaching status of a hospital, or hospital size did not show any association with all-cause 30-day readmission after the index hospitalization. The adjusted Cox-proportional hazard model met all assumptions, including the assumption of linearity and proportional hazards assumption (Schoenfeld proportionality test, P > 0.05). A separate t-test was then used to compare the mean age of those patients who were and were not readmitted (58.07 vs 53.32 years respectively) after the index hospitalization (mean difference: 4.75, 95%CI: 0.75-8.75, P = 0.02). 

Among patients who were readmitted within 30 days, it was noted in exploratory analysis that recurrent esophageal variceal bleeding occurred among 13.3% of patients who underwent TIPS vs 50% among patients who underwent EVB only during the index hospitalization, with RR 0.27 (95%CI: 0.10 - 0.72, p= 0.003). 

## Discussion

This study found no difference in all-cause 30-day readmission rates between patients who underwent either TIPS or EVB only for recurrent esophageal variceal bleeding. In an exploratory analysis, it was noted that the rate of recurrent esophageal variceal bleeding among patients who were readmitted within 30 days was lower in the TIPS group when compared to the EVB group (13.3% vs 50% respectively). Although it was not statistically significant, the inpatient mortality rate during the index hospitalization appeared to be lower in the TIPS group when compared to the EVB group (4.2% vs 10.08% respectively). Patients who underwent TIPS were hospitalized three days longer than patients who underwent EVB only. The most common cause of cirrhosis was alcohol use (59.24%), followed by chronic hepatitis C infection with simultaneous use of alcohol (17.65%), and non-alcoholic steatohepatitis (7.98%). Ascites was the common complication in both groups. The teaching status of the hospital or hospital size during the index hospitalization had no impact on all-cause inpatient mortality during the index hospitalization or on the all-cause 30-day readmission rates. 

The findings of this study mostly correlate with the established literature. In their randomized clinical trial, Pomier-Layrargues et al. found that TIPS did not increase the two-year survival rate compared with variceal band ligation after variceal bleeding [13]. In contrast, in another randomized control trial by García-Pagán et al., early TIPS (within 72 hours of randomization) was found to be associated with better survival at one year when compared to patients who received propranolol or nadolol and long-term endoscopic band ligation (86% vs 61% respectively) [14]. In a recent open-label randomized clinical trial by Lv et al., it was observed that transplantation-free survival at six weeks was 99% in the early TIPS group (within 72 hours) compared with 84% (p=0.02) in the control group (who received the standard treatment of vasoactive drugs, propranolol plus endoscopic band ligation) and at one year, the transplantation-free survival was 86%in the early TIPS group versus 73% in the control group (p=0.046) [15]. In a Cochrane review by Simonetti et al., which has included more than 20 randomized control trials in their review, the conclusion was uncertain whether TIPS versus endoscopic interventions with or without medical treatment reduces all-cause mortality (RR 0.99, 95%CI 0.86-1.13) [12]. However, the follow-up time in their review for mortality was 32.9 months (range 11.7-98). From this aforementioned literature and the findings of our study, it can be deduced that the overall all-cause mortality benefits from TIPS when compared to EVB only is relatively modest and more noticeable within the first 6-12 months of the TIPS procedure. Therefore, TIPS can be considered as a bridge to transplant within the first 6-12 months but there is no convincing mortality benefit of TIPS after one year based on the current evidence. As was noted in our study, TIPS has been associated with a lower risk of recurrent variceal bleeding in most of the prospective clinical trials and reviews [12-14,16,17]. 

Rush et al., in their study using the 2012-2014 NRD sample, found that the 90-day readmission rates due to recurrent esophageal bleeding were lower among patients who underwent TIPS but TIPS did not reduce the overall admission rates [18]. These findings align exactly with the findings of our study that TIPS reduces the incidence of recurrent esophageal bleeding but does not change the 30-day all-cause readmission rates. No comparison has been provided in their studies (no controls) but Vozzo et al. [19] and Khan et al. [20] report the 30-day readmission rate after TIPS to be 36% and 27.81%, respectively. This is close to the 30-day all-cause readmission rate after TIPS in our study, which is 26.32%.

There were limitations to this study. This study was based on the HCUP-NRD, which does not provide data on vitals, laboratory values, or medication administration. We expected some variability in billing codes for the same diagnosis between different providers; for example, hypertension may be billed as I10 or I16. To counter this shortcoming, we used meticulously defined computable phenotypes, as detailed in the Appendices. The HCUP-NRD also does not provide data on out-of-hospital mortality.

## Conclusions

TIPS does not reduce all-cause 30-day readmission rates but is associated with reduced 30-day readmission rates secondary to recurrent esophageal variceal bleeding. TIPS is associated with lengthier hospital stays. However, it is also associated modest survival advantage during index hospitalization when compared to EVB only. From the findings of our study, it can be deduced that if the goal is to reduce all-cause 30-day readmission rates among patients who present with recurrent esophageal variceal bleeding, then TIPS will not be helpful. However, if the goal is to use TIPS to increase the probability of transplant-free survival time and use it as a bridge to liver transplantation within one year, then TIPS can certainly be considered.

## Appendices

International Classification of Diseases, 10th Revision (ICD-10) codes were used to define the computable phenotypes for cirrhosis, esophageal variceal bleeding, esophageal variceal banding (EVB), transjugular intrahepatic portosystemic shunt (TIPS) procedure, baseline comorbidities (such as hypertension), chronic anticoagulation or common comorbidities that require anticoagulation, causes of cirrhosis and complications of cirrhosis. A description of each element is given below. 

ICD-10 codes were divided into two types, specific and broad. A specific code means that it is a billable code and doesn’t require any further details. Broad code means that it is a parent/general code and is non-specific. Many other specific codes fall under the broad code. For example: K70.3 is a more general/broad code for alcoholic cirrhosis of the liver. This is a non-billable code because it is not specific, as it doesn’t provide any details regarding the presence or absence of ascites. Two more specific codes fall under this broad code, which are K70.30 and K70.31. K70.30 represents alcoholic cirrhosis of the liver without ascites, while K70.31 represents alcoholic cirrhosis of the liver with ascites. 

If we needed all of the specific codes that fall under a broad code, for efficiency, we used broad ICD-10 code and that is why we divided the ICD-10 codes into these two categories for our study. For example: We needed ICD-10 codes to identify patients with chronic kidney disease (CKD) or end-stage renal disease (ESRD) status. Instead of using specific ICD-10 codes for each stage of CKD (such as N18.1 for CKD stage-1 or N18.2 for CKD stage-2), we used the broad code of N18 in our search and it captured all forms of CKD as well as ESRD status.

We used ‘dplyr’ package in R-software version 4.3.3 (R Foundation for Statistical Computing, Vienna, Austria) to extract data from the Healthcare Cost and Utilization Project - National Readmission Database (HCUP-NRD) 2019 in this study, using definitions for each element as further detailed below. 

Cirrhosis

If a patient had any of the ICD-10 codes listed in Table 2 during each hospitalization, then it was considered that the patient had a diagnosis of cirrhosis. For example: Mr. A was hospitalized in January 2019 and there was a billing code for K70.30 during the same hospitalization. Mr. A was admitted again in May 2019 and there was a billing code for K70.31 during his second hospitalization. Mr. A had his third hospitalization in August 2019 and during his third hospitalization, he had a billing code for K70.31 as well. In this scenario, Mr. A was admitted to the hospital three times in 2019, and during each hospitalization, there was at least one billing code for cirrhosis (from Table 2). So, Mr. A was identified to have a diagnosis of cirrhosis. 

**Table 2:** ICD-10 diagnostic codes used for cirrhosis ICD-10: International Classification of Diseases, 10th Revision

S. No	Condition	ICD-10 Code	Code Type	Description
1	Cirrhosis	K70.30	Specific	Alcoholic cirrhosis of liver without ascites
		K70.31	Specific	Alcoholic cirrhosis of liver with ascites
		K71.7	Specific	Toxic liver disease with fibrosis and cirrhosis of liver
		K74.3	Specific	Primary Biliary Cholangitis/Cirrhosis
		K74.4	Specific	Secondary biliary cirrhosis
		K74.5	Specific	Biliary cirrhosis, unspecified
		K74.6	Broad	Other and unspecified cirrhosis of liver
		K76.1	Specific	Chronic passive congestion of liver - Applicable to cardiac cirrhosis

In the second scenario, Ms. B was admitted to the hospital in March 2019 and had a billing diagnosis of K74.6. Ms. B was admitted again in May 2019 but during the second hospitalization, there was no billing code for cirrhosis (Table 2). In this second scenario, Ms. B was not considered to have cirrhosis due to inconsistent diagnostic codes between her hospitalizations. 

The above definition for cirrhosis is less sensitive but more specific. This was done with the purpose of more accurately identifying the true study population and to minimize the risk of false flagging. 

Esophageal variceal bleeding

Esophageal variceal bleeding was defined/identified if patient had an ICD-10 diagnostic code of I85.01 or I85.11 among the top 20 diagnostic codes during a hospitalization in 2019.   

EVB

EVB was defined/identified if a patient had an ICD-10 procedure code of 06L34, 06L37, or 06L38 among the top 10 procedure codes during a hospitalization in 2019.   

TIPS

TIPS procedure was defined/identified if patient had an ICD-10 procedure code of 06183J4 or 06184J4 among the top 10 procedure codes during a hospitalization in 2019.

Baseline comorbidities

Baseline comorbidities that have been included in this study are congestive heart failure, ischemic heart disease, hypertension, hyperlipidemia, diabetes mellitus, CKD/ESRD status, chronic obstructive pulmonary disease (COPD)/emphysema, and asthma. 

The presence of each baseline comorbidity was defined by the presence of any ICD-10 diagnostic code from Table 3 during any hospitalization in 2019. For example: Ms. D was hospitalized in January 2019, May 2019, and then again in September 2019. Only during the May 2019 hospitalization, Ms. D had a billing diagnostic code of I10. Ms. D didn’t have any of the billing diagnostic codes for hypertension (as listed in Table 3) during her hospitalization in January 2019 and September 2019. In this case, Ms. D was considered to have hypertension because she had a billing code for hypertension at least during one hospitalization in 2019. 

**Table 3:** ICD-10 diagnostic codes for baseline comorbidities ICD-10: International Classification of Diseases, 10th Revision

S. No	Condition	ICD-10 Code	Code Type	Description
1	Congestive heart failure	I50	Broad	Heart failure
		I11.0	Specific	Hypertensive heart disease with heart failure
		I13.0	Specific	Hypertensive heart and chronic kidney disease with heart failure and stage 1 through stage 4 chronic kidney disease, or unspecified chronic kidney disease
		I13.2	Specific	Hypertensive heart and chronic kidney disease with heart failure and with stage 5 chronic kidney disease, or end stage renal disease
2	Ischemic heart disease	I25.1	Broad	Atherosclerotic heart disease of native coronary artery
		I25.2	Specific	Old myocardial infarction
		I25.5	Specific	Ischemic cardiomyopathy
		I25.6	Specific	Silent myocardial ischemia
		I25.7	Broad	Atherosclerosis of coronary artery bypass graft(s) and coronary artery of transplanted heart with angina pectoris
		I25.8	Broad	Other forms of chronic ischemic heart disease
		I25.9	Specific	Chronic ischemic heart disease, unspecified
		I21.0	Broad	ST elevation (STEMI) myocardial infarction of anterior wall
		I21.1	Broad	ST elevation (STEMI) myocardial infarction of inferior wall
		I21.2	Broad	ST elevation (STEMI) myocardial infarction of other sites
		I21.3	Specific	ST elevation (STEMI) myocardial infarction of unspecified site
		T82.855	Broad	Stenosis of coronary artery stent
		T82.21	Broad	Mechanical complication of coronary artery bypass graft
		Z95.5	Specific	Presence of coronary angioplasty implant and graft
3	Hypertension	I10	Specific	Essential (primary) hypertension
		I11	Broad	Hypertensive heart disease
		I12	Broad	Hypertensive chronic kidney disease
		I13	Broad	Hypertensive heart and chronic kidney disease
		I16	Broad	Hypertensive crisis
4	Diabetes mellitus	E10	Broad	Type 1 diabetes mellitus
		E11	Broad	Type 2 diabetes mellitus
5	Hyperlipidemia	E78.0	Broad	Pure hypercholesterolemia
		E78.1	Specific	Pure hyperglyceridemia
		E78.2	Specific	Mixed hyperlipidemia
		E78.49	Specific	Other hyperlipidemia
		E78.5	Specific	Hyperlipidemia, unspecified
6	Chronic kidney disease / End-stage renal disease	I12	Broad	Hypertensive chronic kidney disease
		I13	Broad	Hypertensive heart and chronic kidney disease
		E10.22	Specific	Type 1 diabetes mellitus with diabetic chronic kidney disease
		E11.22	Specific	Type 2 diabetes mellitus with diabetic chronic kidney disease
		N18	Broad	Chronic kidney disease
7	Chronic obstructive pulmonary disease / Emphysema	J43	Broad	Emphysema
		J44	Broad	Other chronic obstructive pulmonary disease
8	Asthma	J45	Broad	Asthma
9	Pulmonary hypertension	I27.0	Specific	Primary pulmonary hypertension
		I27.2	Broad	Other secondary pulmonary hypertension
10	Tricuspid valve regurgitation	I36.1	Specific	Nonrheumatic tricuspid (valve) insufficiency
		I36.2	Specific	Nonrheumatic tricuspid (valve) stenosis with insufficiency
		I07.1	Specific	Rheumatic tricuspid insufficiency
		I07.2	Specific	Rheumatic tricuspid stenosis and insufficiency
11	Polycystic liver disease	Q44.6	Specific	Cystic disease of liver

If the patient has many chronic comorbidities and they are stable, sometimes clinicians don’t bill for all comorbidities. In contrast to the definition of cirrhosis (as mentioned above), it was the reason why a more sensitive but lesser specific criteria was used to define each baseline comorbidity. For instance: same Ms. D had a history of cirrhosis, congestive heart failure, ischemic heart disease, CKD, hyperlipidemia, and hypertension. Ms. D was admitted for acute kidney injury on CKD each time in 2019. Ms. D's blood pressure was within normal range with only one anti-hypertensive medication during all of her three hospitalizations in 2019. A clinician didn’t bill for hypertension in January 2019 and September 2019 but another clinician decided to bill for it in May 2019. This way, Ms. D ended up having a billing code for hypertension only in May 2019. In this scenario, Ms. D was considered to have hypertension in our study.

Anticoagulation or comorbidities that may require anticoagulation

ICD-10 diagnostic codes for chronic anticoagulation and common conditions that require anticoagulation are given in Table 4. If a patient had any of the diagnostic codes present in Table 4 during the index hospitalization or any hospitalization before the index hospitalization in 2019, then such a patient was considered/presumed to be on anticoagulation (which independently increases the risk of bleeding).

**Table 4:** ICD-10 diagnostic codes for chronic anticoagulation and common conditions that require anticoagulation ICD-10: International Classification of Diseases, 10th Revision

S. No	Condition	ICD-10 Code	Code Type	Description
1	Chronic anticoagulation	Z79.01	Specific	Long-term (current) use of anticoagulants
2	Atrial fibrillation and flutter	I48	Broad	Atrial fibrillation and flutter
3	Deep vein thrombosis	I82.4	Broad	Acute embolism and thrombosis of deep veins of lower extremity
		I82.5	Broad	Chronic embolism and thrombosis of deep veins of lower extremity
		I82.6	Broad	Acute embolism and thrombosis of veins of upper extremity
		I82.7	Broad	Chronic embolism and thrombosis of veins of upper extremity
		I82.A	Broad	Embolism and thrombosis of axillary vein
		I82.B	Broad	Embolism and thrombosis of subclavian vein
		I82.C	Broad	Embolism and thrombosis of internal jugular vein
4	Pulmonary embolism	I26	Broad	Pulmonary embolism
5	Portal vein thrombosis	I81	Specific	Portal vein thrombosis

Causes of cirrhosis

The cause of cirrhosis was identified via the presence of any ICD-10 diagnostic code from Table 5 during any hospitalization in 2019.

**Table 5:** ICD-10 diagnostic codes for causes of cirrhosis ICD-10: International Classification of Diseases, 10th Revision

S. No	Condition	ICD-10 Code	Code Type	Description
1	Chronic Hepatitis B	B18.0	Specific	Chronic viral hepatitis B with delta-agent
		B18.1	Specific	Chronic viral hepatitis B without delta-agent
		B19.10	Specific	Viral hepatitis B without hepatic coma
		B19.11	Specific	Viral hepatitis B with hepatic coma
2	Chronic Hepatitis C	B18.2	Specific	Chronic viral hepatitis C
		B19.20	Specific	Viral hepatitis C without hepatic coma
		B19.21	Specific	viral hepatitis C with hepatic coma
3	Alcohol Use	K70.30	Specific	Alcoholic cirrhosis of liver without ascites
		K70.31	Specific	Alcoholic cirrhosis of liver with ascites
4	Cardiac	K76.1	Specific	Chronic passive congestion of liver - Applicable to cardiac cirrhosis
5	Hereditary hemochromatosis	E83.110	Specific	Hereditary hemochromatosis
6	Autoimmune hepatitis	K75.4	Specific	Autoimmune hepatitis
7	Nonalcoholic steatohepatitis	K75.81	Specific	Nonalcoholic steatohepatitis
8	Primary biliary cholangitis	K74.3	Specific	Primary biliary cholangitis, previously called primary biliary cirrhosis
9	Primary sclerosing cholangitis	K83.01	Specific	Primary sclerosing cholangitis
10	Cystic fibrosis	E84.8	Specific	Cystic fibrosis with other manifestations
11	Wilson’s Disease	E83.01	Specific	Wilson’s Disease

Complications related to cirrhosis

Only the index hospitalization was assessed for the presence of ICD-10 codes listed in Table 6 to identify each complication.

**Table 6:** ICD-10 codes for cirrhosis complications during index hospitalization ICD-10: International Classification of Diseases, 10th Revision

S. No	Condition	ICD-10 Code	Code Type	Description
1	Spontaneous bacterial peritonitis	K65.2	Specific	Spontaneous bacterial peritonitis
2	Encephalopathy	K76.82	Specific	Hepatic encephalopathy
		G93.4	Broad	Other and unspecified encephalopathy
3	Hepatorenal syndrome	K76.7	Specific	Hepatorenal syndrome
4	Ascites	R18	Broad	Ascites
		K70.31	Specific	Alcoholic cirrhosis of liver with ascites
5	Hepatocellular carcinoma	C22.0	Specific	liver cell carcinoma
6	Red blood cells transfusion	30233N1	Specific	Transfusion of non-autologous red blood cells into peripheral vein, percutaneous approach
		30243N1	Specific	Transfusion of non-autologous red blood cells into central vein, percutaneous approach
